# Supplementary material for: Single-cell transcriptome analysis of epithelial, immune, and stromal signatures and interactions in human ovarian cancer
Source: Commun Biol. 2024 Jan 26;7:131. doi: 10.1038/s42003-024-05826-1 (PMC10817929; doi:10.1038/s42003-024-05826-1)
Supplement: Supplementary file 2 — Supplementary Information [file 42003_2024_5826_MOESM2_ESM.pdf]

Supplementary Information

Single-cell transcriptome analysis of epithelial, immune, and stromal signatures and interactions in human ovarian cancer

Chaochao Chai[1, 2, 3*], Langchao Liang[1, 2, 3*], Nanna S. Mikkelsen[4], Wei Wang[5], Wandong Zhao[1,2], Chengcheng Sun[1,2], Rasmus O. Bak[4], Hanbo Li[2,3], Lin Lin[4,6], Fei Wang[2, 3, 4‡], Yonglun Luo[2, 3, 4, 6‡]

Affiliations

[1]College of Life Sciences, University of Chinese Academy of Sciences, Beijing 10049, China.

[2]Lars Bolund Institute of Regenerative Medicine Qingdao-Europe Advanced Institute for LifeScience, BGI Research, Qingdao 266555, China.

[3]BGI Research, Shenzhen 518083, China.

[4]Department of Biomedicine, Aarhus University, Aarhus, Denmark.

[5]Department of Obstetrics and Gynecology, Tongji Hospital, Tongji Medical College, Huazhong University of Science and Technology, Wuhan, China.

[6]Steno Diabetes Center Aarhus, Aarhus University Hospital, Aarhus, Denmark.

*These authors contributed equally.

‡ These authors jointly supervised this work.

Corresponding should be addressed to Fei Wang (wangfei@biomed.au.dk), and Yonglun Luo (alun@biomed.au.dk).

Supplementary Figure 1:

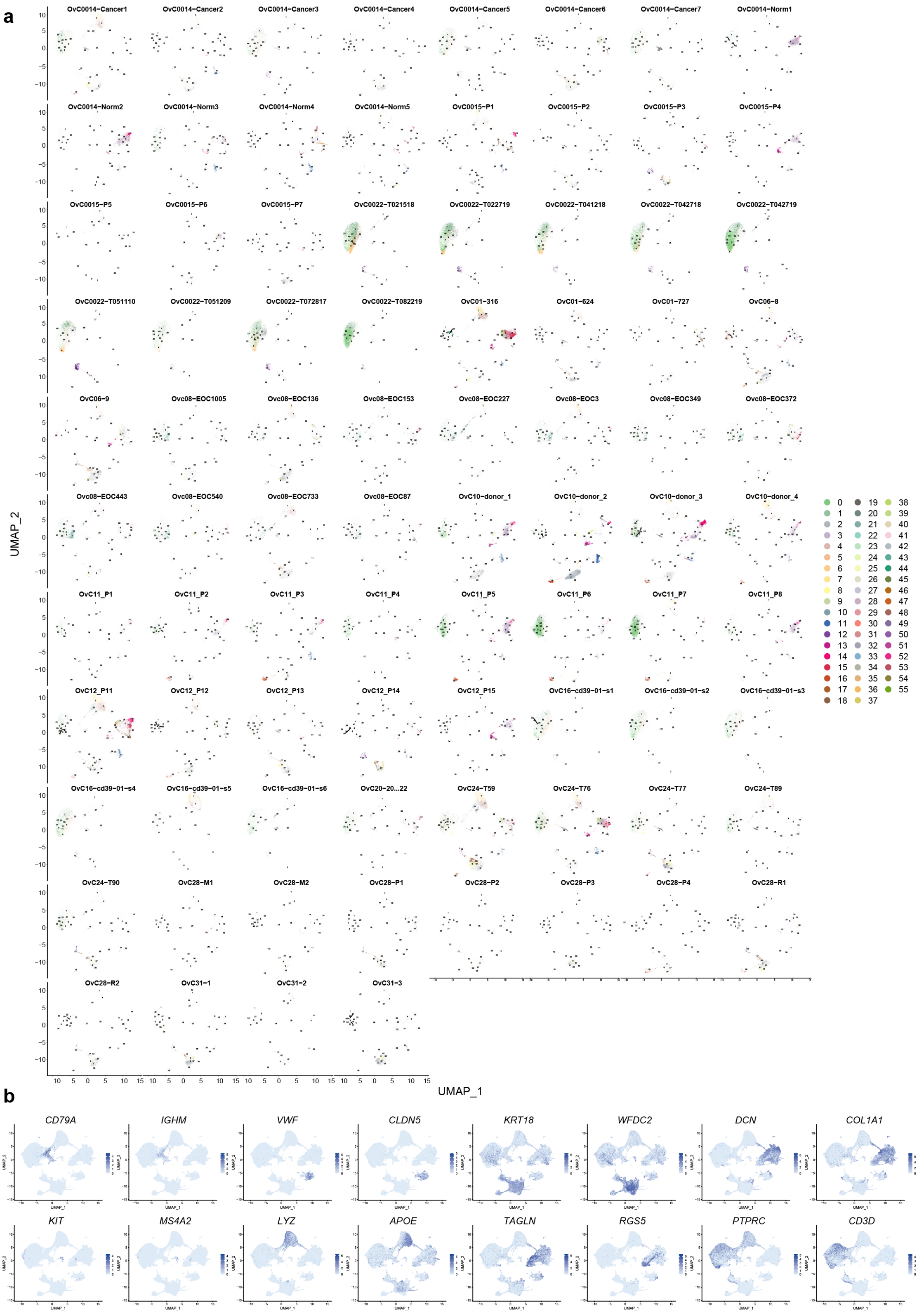

a The UMAP plot is separated by patient samples, with colors indicate different clusters.

b The UMAP plot color coded for the expression of marker genes for major cell type.

Supplementary Figure 2:

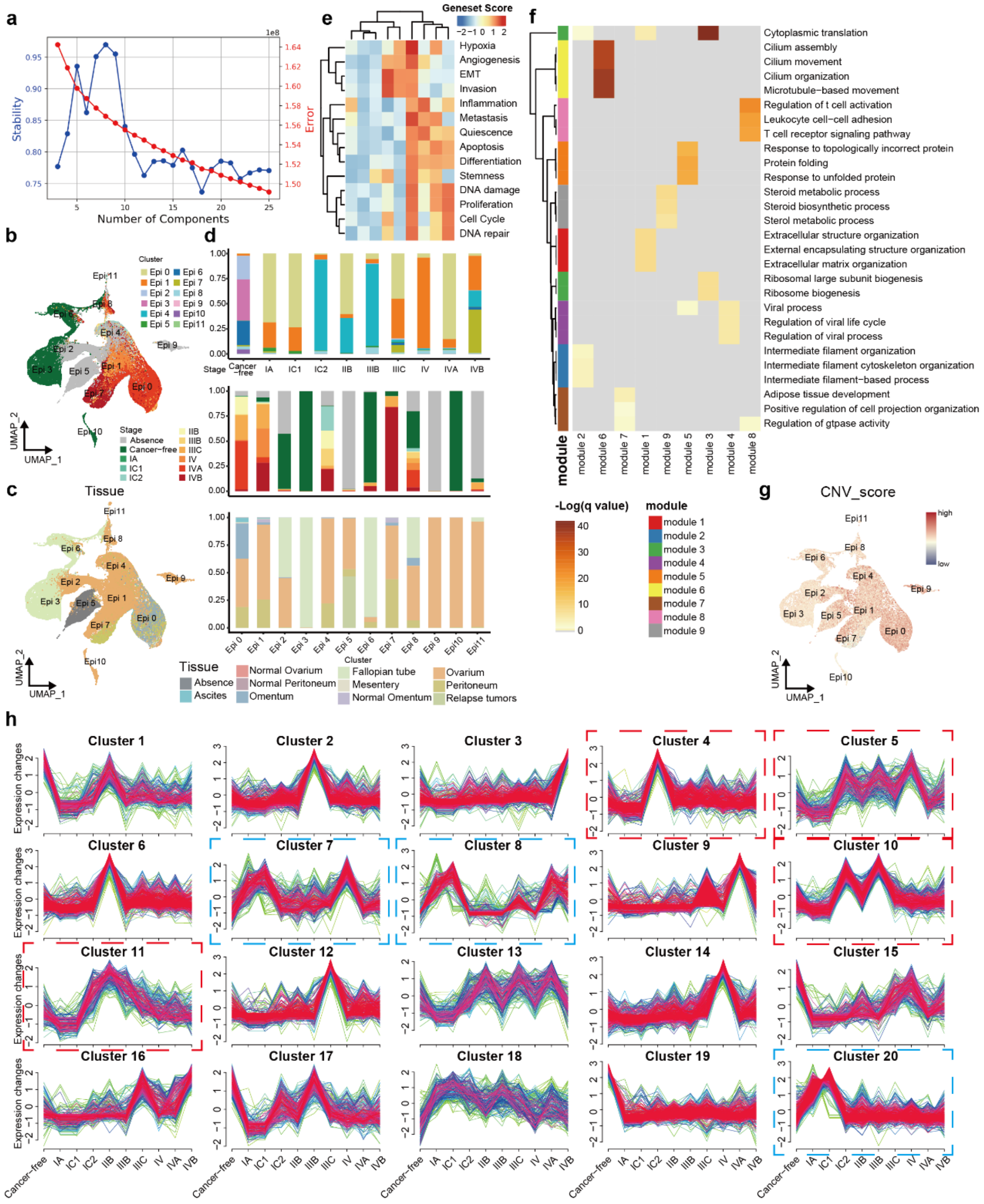

a The curve chart shows the stability and error of different quantity of gene module. **b** The UMAP plot shows different stages of the epithelial cell by color. **c** The UMAP plot shows different tissues of the epithelial cell by color. **d** Stacked bar graph of the proportion of epithelial cell cluster in each stage and the proportion of stage and tissue in each epithelial cell sub cluster. **e** Heat map show the GSVA scores of cancer-related function gene sets for each gene module. **f** Heat map show the GO enrichment terms of the top 200 contribution genes of each gene module. **g** The UMAP plot shows CNV

score of the epithelial cell by color. h Clustering analysis based on similar temporal patterns of high variation genes in epithelial cells.

Supplementary Figure 3:

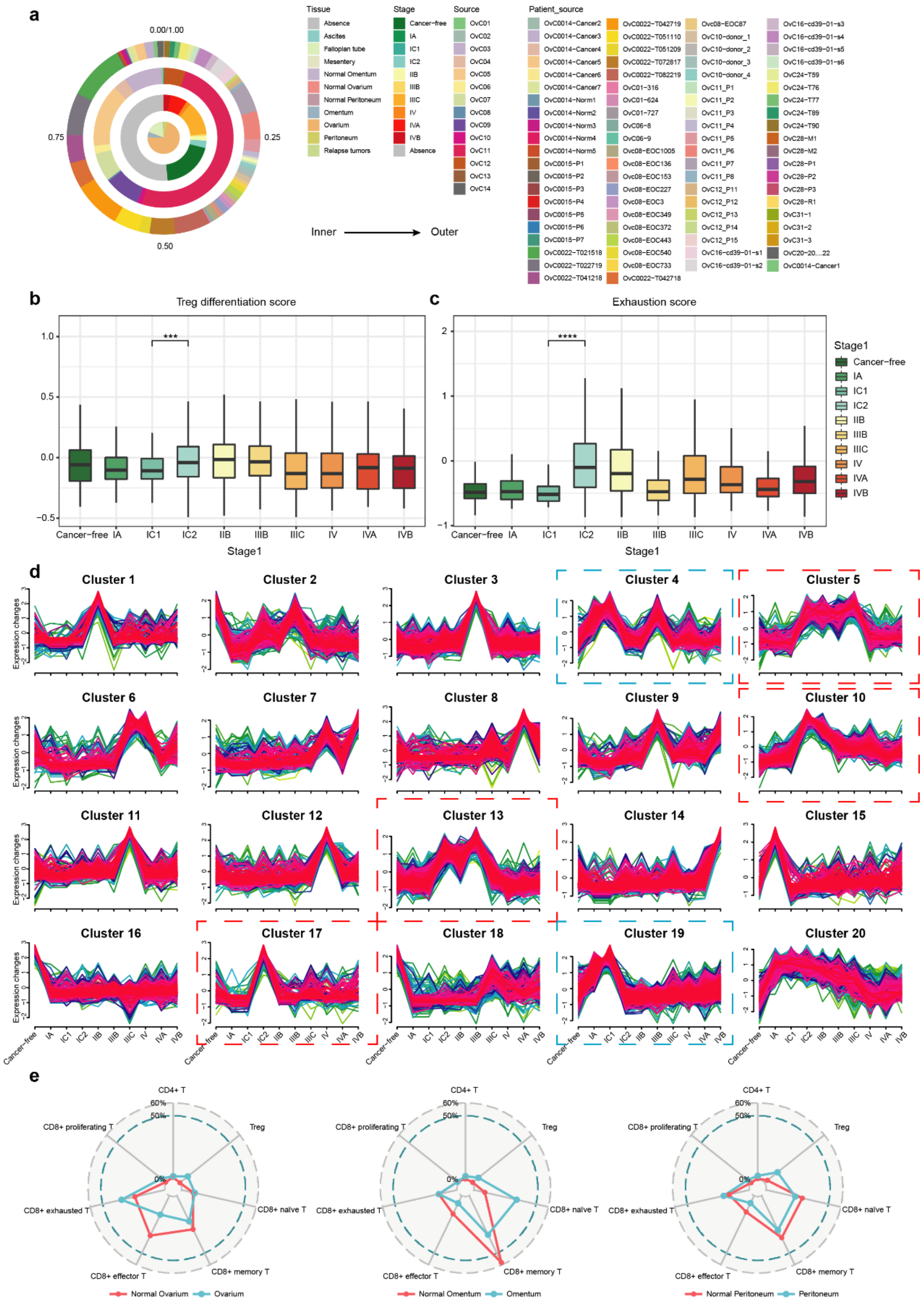

a The donut diagram shows the different tissue, stage, source, patient sample composition of the T cell. **b** The box plot shows change of Treg differentiation score in T cell with stage (Total 35,499 single cells). **c** The box plot shows change of exhaustion

differentiation score in T cell with stage (Total 35,499 single cells). *, $P < 0.05$; **, $P < 0.01$; ***, $P < 0.001$, ****, $P < 0.0001$. **d** Clustering analysis based on similar temporal patterns of high variation genes in T cells. **e** The radar map shows changes in the proportion of T cell subtypes in ovarium, omentum, peritoneum versus normal tissue. The red dashed box indicates increase from stage IC1 to stage IC2, and the blue dashed box indicates a decrease.

Supplementary Figure 4:

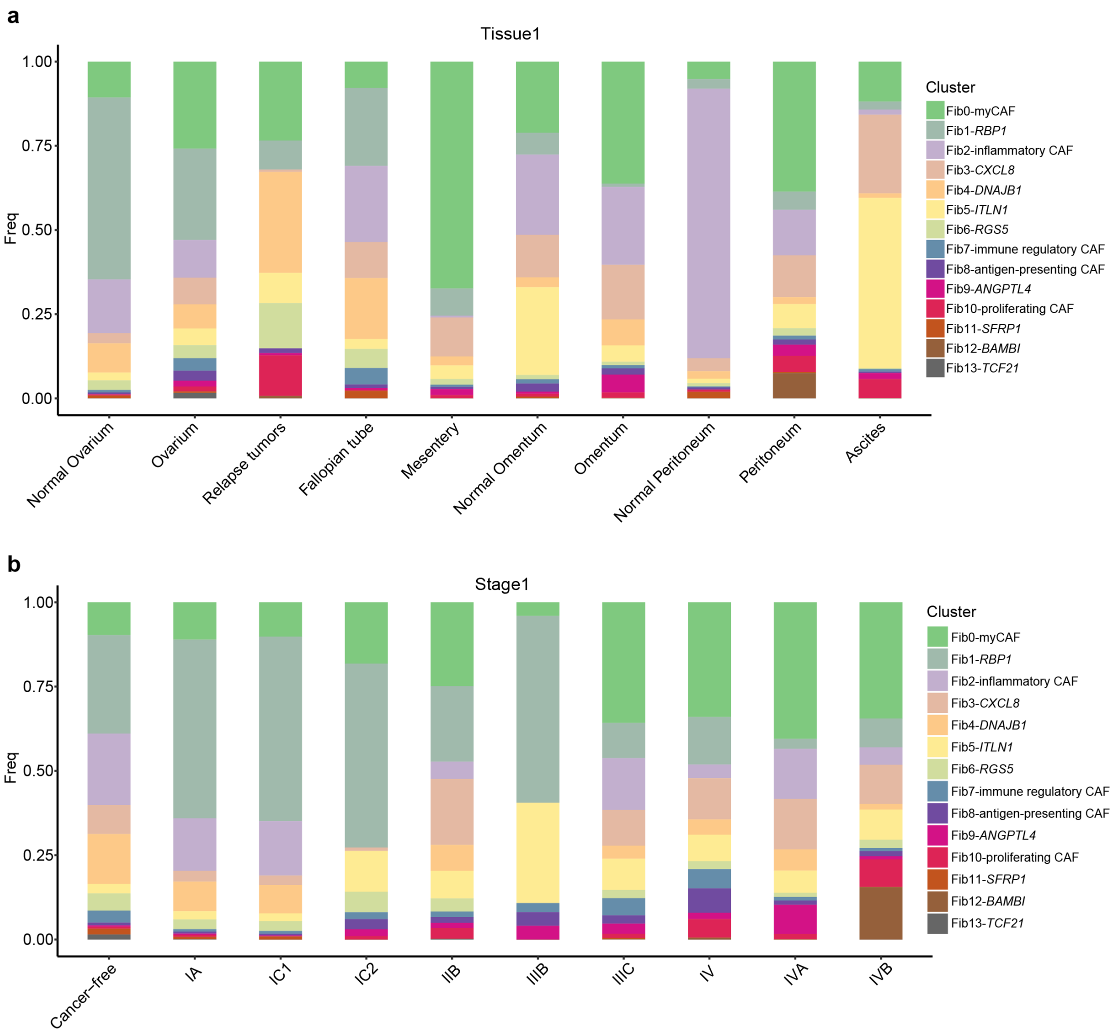

a Stacked bar graph of the proportion of fibroblast subtypes in each tissue. **b** Stacked bar graph of the proportion of fibroblast subtypes in each stage.

Supplementary Figure 5:

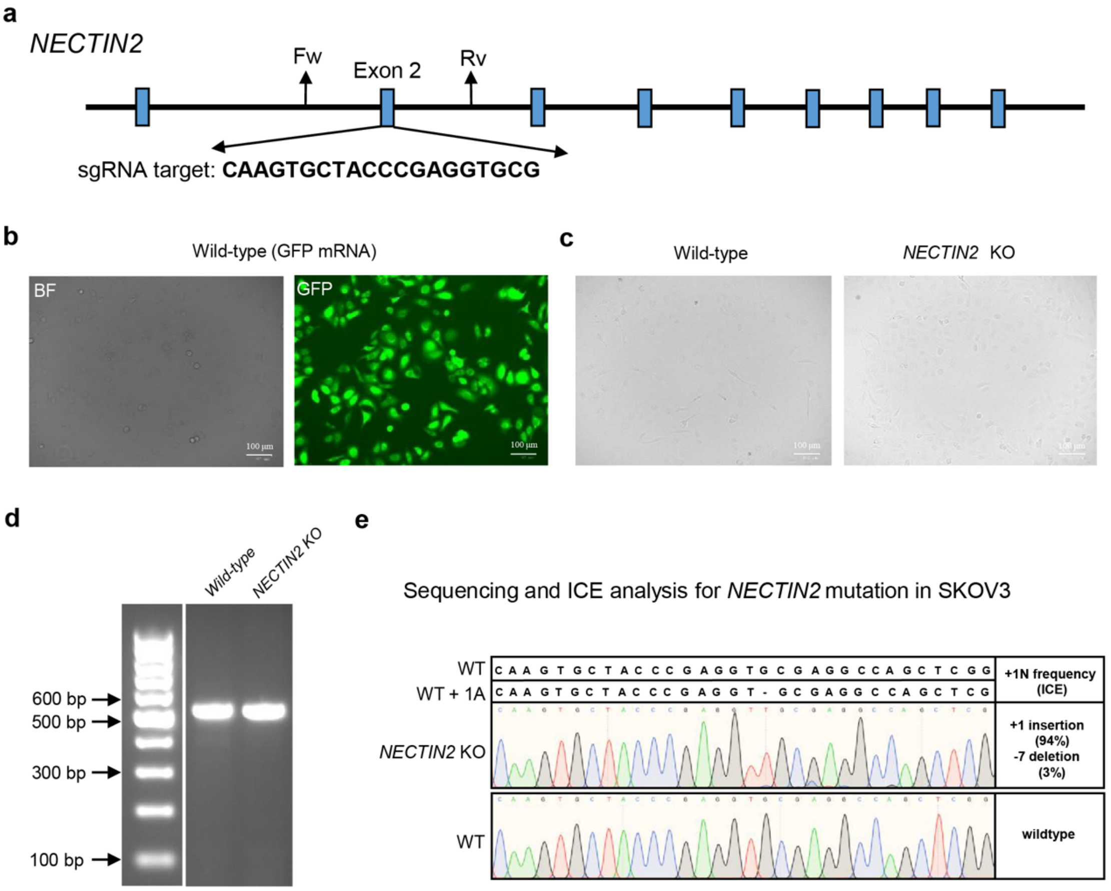

a sgRNA targeting exon 2 of the *NECTIN2* gene and primers sites. **b** EGFP mRNA transduction efficiency in SKOV3 cells. **c** Cell morphology of SKOV3 and *NECTIN2* knock-out SKOV3 cells. **d** Gel images of PCR amplicons for wildtype and *NECTIN2* knock out target regions. **e** Sequences and ICE analysis for *NECTIN2* mutation in SKOV3 cells.
