# Supplementary material for: Single-cell transcriptome analysis of epithelial, immune, and stromal signatures and interactions in human ovarian cancer
Source: Commun Biol. 2024 Jan 26;7:131. doi: 10.1038/s42003-024-05826-1 (PMC10817929; doi:10.1038/s42003-024-05826-1)
Supplement: Supplementary file 3 — Description of Additional Supplementary Files [file 42003_2024_5826_MOESM3_ESM.pdf]

Description of Additional Supplementary Files

File name: Supplementary Data 1
Description: Literature collection and summary and the source data of Figure 1.

File name: Supplementary Data 2
Description: The source data of Figure 2.

File name: Supplementary Data 3
Description: T cell exhaustion and regulatory T cell differentiation gene set.

File name: Supplementary Data 4
Description: The source data of Figure 4.

File name: Supplementary Data 5
Description: The source data of Figure 5.

File name: Supplementary Data 6
Description: Nectin 2 sgRNA and Oligo sequences.
