# Supplementary material for: Single-cell transcriptome analysis of epithelial, immune, and stromal signatures and interactions in human ovarian cancer
Source: Commun Biol. 2024 Jan 26;7:131. doi: 10.1038/s42003-024-05826-1 (PMC10817929; doi:10.1038/s42003-024-05826-1)
Supplement: Supplementary file 10 — Reporting Summary [file 42003_2024_5826_MOESM10_ESM.pdf]

nature portfolio

Corresponding author(s): Fei Wang, Yonglun Luo

Last updated by author(s): Nov 23, 2023

Reporting Summary

Nature Portfolio wishes to improve the reproducibility of the work that we publish. This form provides structure for consistency and transparency in reporting. For further information on Nature Portfolio policies, see our Editorial Policies and the Editorial Policy Checklist.

Statistics

For all statistical analyses, confirm that the following items are present in the figure legend, table legend, main text, or Methods section.

n/a	Confirmed	
☐	☒	The exact sample size (*n*) for each experimental group/condition, given as a discrete number and unit of measurement
☐	☒	A statement on whether measurements were taken from distinct samples or whether the same sample was measured repeatedly
☐	☒	The statistical test(s) used AND whether they are one- or two-sided<br>*Only common tests should be described solely by name; describe more complex techniques in the Methods section.*
☒	☐	A description of all covariates tested
☐	☒	A description of any assumptions or corrections, such as tests of normality and adjustment for multiple comparisons
☐	☒	A full description of the statistical parameters including central tendency (e.g. means) or other basic estimates (e.g. regression coefficient) AND variation (e.g. standard deviation) or associated estimates of uncertainty (e.g. confidence intervals)
☐	☒	For null hypothesis testing, the test statistic (e.g. *F*, *t*, *r*) with confidence intervals, effect sizes, degrees of freedom and *P* value noted<br>*Give P values as exact values whenever suitable.*
☒	☐	For Bayesian analysis, information on the choice of priors and Markov chain Monte Carlo settings
☒	☐	For hierarchical and complex designs, identification of the appropriate level for tests and full reporting of outcomes
☒	☐	Estimates of effect sizes (e.g. Cohen's *d*, Pearson's *r*), indicating how they were calculated

Our web collection on statistics for biologists contains articles on many of the points above.

Software and code

Policy information about availability of computer code

Data collection	The data used in this study are all from published 14 articles, and the specific accession numbers and article information have been provided in Supplementary Table 1.
Data analysis	We used R package harmony(version 0.1.0) to remove batch effects between samples.R package org.Hs.eg.db (version 3.16.0) to convert the symbol and entrezid. GO enrichment analysis was performed using the clusterProfiler(version 4.6.0).We applied GSVA (version 1.46.0) for scoring analysis of functional gene sets in tumor cells and top10 marker gene of myCAF. We then used pheatmap (version 1.0.12) for visualization.We used the Monocle2(version 2.22.0) to explore the underlying changes in epithelial cell function and identify potential lineage differentiation and monocle3 (version 1.2.9) to explore the differentiation trajectory of CD8T cell subtypes. We used inferCNV (version 1.10.1) R package to estimate CNV level in malignant epithelial cell. R package SCENT (Version 1.0.3) was used to estimate the epithelial cell differentiation potency. We used Mfuzz (Version 2.54.0) to identify time dependent transcriptional program in epithelial cell, T cell and fibroblast. The R package cgdsr (version 1.3.0) was used to link to the cBioPortal database (http://www.cbioportal.org/) and download the TCGA-OV data (ov_tcga_pan_can_atlas_2018). We used CellChat(version 1.5.0) to perform ligand-receptor interaction analysis.

For manuscripts utilizing custom algorithms or software that are central to the research but not yet described in published literature, software must be made available to editors and reviewers. We strongly encourage code deposition in a community repository (e.g. GitHub). See the Nature Portfolio guidelines for submitting code & software for further information.

Data

Policy information about availability of data

All manuscripts must include a data availability statement. This statement should provide the following information, where applicable:

- Accession codes, unique identifiers, or web links for publicly available datasets
- A description of any restrictions on data availability
- For clinical datasets or third party data, please ensure that the statement adheres to our policy

Data Availability Statement:
The data used in this study are all from published articles, and the specific accession numbers and article information have been provided in Supplementary Table 1. These data have been publicly released and can be accessed and obtained from the corresponding journals or databases. Additionally, we have created an interactive website (https://dreamapp.biomed.au.dk/OvaryCancer_DB/) for the visualization of the datasets. Source data underlying Figs. 1d, 2c, 2h, 4d, 5b-d are presented in Supplementary Data 1.2, 1.3, 2.1, 2.5, 4.2, 5.1, 5.2. Other data are available upon reasonable request to the corresponding authors.

Human research participants

Policy information about studies involving human research participants and Sex and Gender in Research.

Reporting on sex and gender	This study focuses on ovarian cancer, and therefore the data collected are all from women. We strictly followed the relevant ethical regulations and laws in our study, and conducted sufficient validation and analysis of the data. During the data analysis process, we did not involve gender-based analysis, but instead treated all data as a whole for analysis.
Population characteristics	This study was designed to investigate molecular changes in different stages of ovarian cancer, regardless of race, ethnicity, or other socially relevant groupings.
Recruitment	This study used data from published articles and did not recruit participants.
Ethics oversight	The data used in this study are all from published articles, and the detailed information is shown in Supplementary Table 1. These articles have obtained ethical approval documents and strictly abide by relevant ethical regulations and laws and regulations.

Note that full information on the approval of the study protocol must also be provided in the manuscript.

Field-specific reporting

Please select the one below that is the best fit for your research. If you are not sure, read the appropriate sections before making your selection.

☒ Life sciences ☐ Behavioural & social sciences ☐ Ecological, evolutionary & environmental sciences

For a reference copy of the document with all sections, see nature.com/documents/nr-reporting-summary-flat.pdf

Life sciences study design

All studies must disclose on these points even when the disclosure is negative.

Sample size	In this study, we collected single-cell transcriptomic datasets of ovarian cancer(OC) encompassing all clinical stages, comprising data from 84 ovarian patients with a total of 505,102 single cells. 40 single cell RNA-seq literature for OC was collected and all data were downloaded as far as possible (Supplementary Table 1). We selected 84 patients data from 10X genomics platforms to avoid batch effects across platforms.
Data exclusions	After obtaining the original single-cell expression matrix, we follow the quality control criteria described in the original article and nFeature_RNA > 500 & nFeature_RNA < 7000 & percent.mt < 25 to filter the data. If the specific quality control conditions are not mentioned in the article, we follow nFeature_RNA > 500 & nFeature_RNA < 7000 & percent.mt < 25 to filter (Table1).
Replication	The results of all analyses in this study are reproducible.
Randomization	All the data in this study are from published articles and have nothing to do with randomness.
Blinding	The data for this project is from published articles and blinding was not relevant to this study.

Reporting for specific materials, systems and methods

We require information from authors about some types of materials, experimental systems and methods used in many studies. Here, indicate whether each material, system or method listed is relevant to your study. If you are not sure if a list item applies to your research, read the appropriate section before selecting a response.

Materials & experimental systems

n/a	Involved in the study
☒	☐ Antibodies
☐	☒ Eukaryotic cell lines
☒	☐ Palaeontology and archaeology
☒	☐ Animals and other organisms
☒	☐ Clinical data
☒	☐ Dual use research of concern

Methods

n/a	Involved in the study
☒	☐ ChIP-seq
☒	☐ Flow cytometry
☒	☐ MRI-based neuroimaging

Eukaryotic cell lines

Policy information about cell lines and Sex and Gender in Research

Cell line source(s)	The SKOV-3 cells were obtained from the American Type Culture Collection (ATCC).
Authentication	*Describe the authentication procedures for each cell line used OR declare that none of the cell lines used were authenticated.*
Mycoplasma contamination	All the cells were tested negative for mycoplasma.
Commonly misidentified lines (See ICLAC register)	No commonly misidentified cell lines were used in this study.
